# Arterial spin labeling image findings in the acute phase in paediatric patients with acute encephalopathy with biphasic seizures and late reduced diffusion

**DOI:** 10.3389/fnins.2023.1252410

**Published:** 2023-09-19

**Authors:** Go Kawano, Kentaro Tokutomi, Yoshitomo Kikuchi, Kensuke Sakata, Hirotaka Sakaguchi, Takaoki Yokochi, Yukihiro Akita, Toyojiro Matsuishi

**Affiliations:** ^1^Department of Paediatrics, St Mary’s Hospital, Fukuoka, Japan; ^2^Department of Radiology, St Mary’s Hospital, Fukuoka, Japan; ^3^Research Centre for Children and Research Centre for Rett Syndrome, St Mary’s Hospital, Fukuoka, Japan; ^4^Division of Gene Therapy and Regenerative Medicine, Cognitive and Molecular Research Institute of Brain Diseases, Kurume University School of Medicine, Fukuoka, Japan

**Keywords:** acute encephalopathy with biphasic seizures and late reduced diffusion, arterial spin labeling, cerebral blood flow, early seizure, late seizures, apparent diffusion coefficient, diffusion-weighted image

## Abstract

**Introduction:**

Diagnosing acute encephalopathy with biphasic seizures and late reduced diffusion (AESD) after the first seizure (early seizure/seizures, ES/ESs) is challenging because a reduced apparent diffusion coefficient (ADC) in the cortical or subcortical white matter, often described as having a “bright-tree appearance (BTA),” is usually not observed until secondary seizures (late seizures, LSs) occur. Previous studies have reported hypoperfusion on arterial spin labeling (ASL) within 24 h after ES/ESs in patients with AESD and hyperperfusion within 24 h after LS onset. This study aimed to investigate cerebral blood flow in the hyperacute phase (between ES/ESs and LSs) using ASL in patients with AESD.

**Methods:**

Eight ASL images were acquired in six patients with AESD admitted to our hospital from October 2021 to October 2022. ASL findings in the hyperacute phase were investigated and video-electroencephalogram findings obtained around ASL image acquisition in the hyperacute phase were evaluated.

**Results:**

Four ASL images were obtained for three patients before LS onset, with three images showing hyperperfusion areas and one image showing hypoperfusion areas. These hyperperfuion regions coincided with BTA on subsequent images of these patients.

In one patient, the first ASL image was obtained in the late hyperacute phase and revealed hyperperfusion areas with a slightly abnormal change on diffusion-weighted image (DWI), which were not accompanied by ADC abnormalities. The second ASL image obtained 51 h after the first ASL, and before LS onset revealed more prominent hyperperfusion areas than the first ASL image, which were accompanied by BTA. In another patient, the ASL image obtained 82 h after ES revealed hyperperfusion areas without abnormal change on DWI or ADC.

**Conclusion:**

This study revealed that two patients exhibited hyperperfusion regions and another patient exhibited hypoperfusion regions among three patients who underwent ASL imaging during the period from 24 h after ES/ESs to LSs in patients with LSs or cooling initiation in patients without LSs due to early anaesthesia induction (late hyperacute phase). Further prospective studies on cerebral blood flow are required to explore the relationship among the timing of image acquisition, the presence of electrographic seizures, and ASL findings in patients with AESD.

## 1. Introduction

Acute encephalopathy with biphasic seizures and late reduced diffusion (AESD) is a type of acute infection-triggered encephalopathy. It is characterized by the occurrence of febrile seizures (early seizure/early seizures, ES/ESs) and secondary seizures (late seizures, LSs), with a reduced apparent diffusion coefficient (ADC) in the cortical or subcortical white matter. This is often described as having a “bright-tree appearance (BTA)” on diffusion-weighted imaging (DWI) images and appears within 9 days of ES/ESs following a transient recovery of consciousness (Takanashi et al., 2006; Hayashi et al., 2012; Yadav et al., 2013). AESD mostly affects children who are younger than school age and has been mainly reported in Japan and East Asia (Hoshino et al., 2012). Some patients with AESD do not manifest LSs because of early interventions, such as continuous administration of sedatives during targeted temperature management (Hayashi et al., 2012; Sakata et al., 2020).

In our previous study, we observed a correlation between worse outcomes and the duration from ES/ESs to the initiation of therapeutic hypothermia in patients with AESD cooled before LSs (Sakata et al., 2020). However, BTA is usually not observed until the occurrence of LSs in patients with AESD, making early diagnosis challenging and leading to delays in intervention for this population. The mechanisms underlying AESD have not been clarified; however, evidence suggests that it may be attributed to late neuronal death triggered by extracellular glutamate stimulation during ES/ESs (Takanashi et al., 2006; Lee et al., 2019).

Arterial spin labeling (ASL) is a magnetic resonance imaging (MRI) sequence used for measuring cerebral blood flow (CBF) non-invasively. It utilizes magnetically labeled blood water as a flow tracer. Compared with single photon emission computed tomography (SPECT), ASL can be easily obtained alongside structural MRI without requiring contrast agents or radioactive tracers. Currently, ASL is widely used for evaluating CBF in various diseases, such as anoxic injury, epilepsy, cerebral vascular diseases, and mild traumatic brain injury (Pollock et al., 2008; Telischak et al., 2015; Haller et al., 2016; Gaxiola-Valdez et al., 2017).

CBF results in patients with seizures were inconsistent owing to variations in time points and brain regions. However, studies using dynamic contrast-enhanced MRI and positron emission tomography discovered elevated CBF during ictus and suppressed CBF during interictal in the ipsilateral temporal lobe of patients with refractory unilateral mesial temporal lobe epilepsy and hippocampal sclerosis. Positron emission tomography hypometabolism and ictal SPECT hypoperfusion were maximal in the ipsilateral frontal lobe (Nelissen et al., 2006). ASL imaging in patients with epileptic convulsions revealed that ictus increased CBF and decreased CBF in the postictal period (Telischak et al., 2015). In addition, hypoperfusion in the seizure onset zone has been reported on ASL during the immediate postictal period (< 60 min) (Gaxiola-Valdez et al., 2017).

Moreover, previous studies have reported hypoperfusion on ASL within 24 h after ES/ESs and hyperperfusion on ASL within 24 h after LSs in patients with AESD (Kuya et al., 2017; Yokoyama et al., 2017; Uetani et al., 2020). These abnormal perfusion regions matched the regions with a reduced ADC observed in the cortical or subcortical white matter during or after LSs. Uetani, et al. speculated that the peri-ictal state and/or loss of autoregulation due to hypoxia caused by status epilepticus convulsions might play important roles in hyperperfusion within 24 h after LSs (Uetani et al., 2020). In a case report of a patient with AESD, MR images obtained a day before the patient started experiencing LSs showed hyperperfusion in bilateral posterior frontal areas on ASL with high signal intensity on DWI images and no ADC abnormalities (Morita et al., 2021).

We aimed to investigate the ASL findings in patients with AESD in our hospital, focusing on the timing of the ASL image acquisition obtained during the hyperacute phase, which is the period between ES/ESs and LSs.

## 2. Materials and methods

### 2.1. Study population

We examined ASL images of six patients with AESD (median age = 15.5 months; age range = 10–21 months; two girls) out of 12 patients admitted to our facility between October 2021 and October 2022. These patients are part of an ongoing prospective hypothermia plus remote ischaemic post-conditioning (RIPoC) efficacy study on AESD (trial number; UMIN000041484). MR images were scheduled to be acquired for each patient when they experienced febrile status epilepticus and prolonged disturbed consciousness lasting more than 6 h after the seizure or when their AESD scores (Tada or Yokochi score) exceeded 4. The Tada score, which consists of seven variables, including consciousness level, age, duration of convulsions, enforcement of mechanical intubation, serum aspartate transaminase (AST), blood glucose, and serum creatinine (Crea) level, is a predictor of AESD for patients with febrile seizures (Tada et al., 2015). The Yokochi score, another predictive score, includes six variables: serum alanine transaminase (ALT), blood glucose, serum Crea, serum ammonia, blood pH, and time until waking (Yokochi et al., 2016).

ASL image acquisition was also added to the MRI protocol when available. Thiamylal was administered as the sedative for all patients during the MR image acquisition. ASL imaging was not performed for the other six patients with AESD owing to technical reasons, such as a busy MRI schedule or lack of staff available to obtain ASL images during the night shift. In the ongoing prospective hypothermia plus RIPoC efficacy study on AESD, RIPoC was concurrently applied within the initiation of therapeutic hypothermia. Thus, this intervention did not affect the ASL images obtained before that time, especially during the hyperacute phase described below. However, the ASL images obtained after that time point may have been influenced by this procedure, and their analysis falls outside the scope of this manuscript.

**Figure 1:**
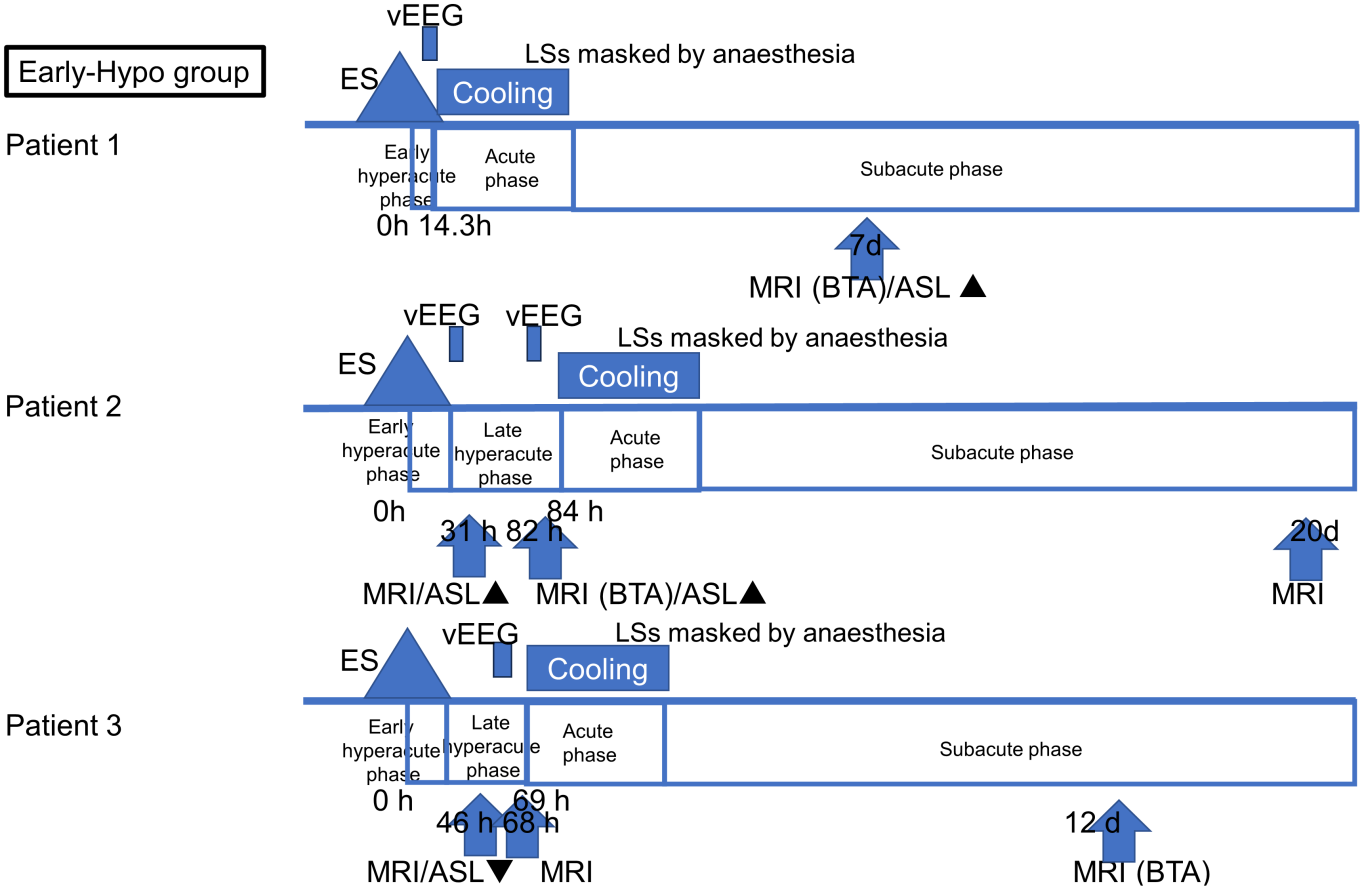
Outlines of the timing of ASL and MR imaging acquisition in each patient in the Early-Hypo group. The clinical course of AESD was divided into four phases: early hyperacute phase (within 24 h after ES/ESs), late hyperacute phase (from 24 h after ES/ESs to cooling initiation due to LS masked by anaesthesia), acute phase (within 3 days after cooling initiation), and subacute phase (from 3 days after cooling initiation to 30 days after ES/ESs. The timing of the initiation of LSs, cooling initiation, and image acquisition is shown in hours from the onset of ES/ESs. ▲ Hyperperfusion in ASL. ▼ Hypoperfusion in ASL.

**Figure 2:**
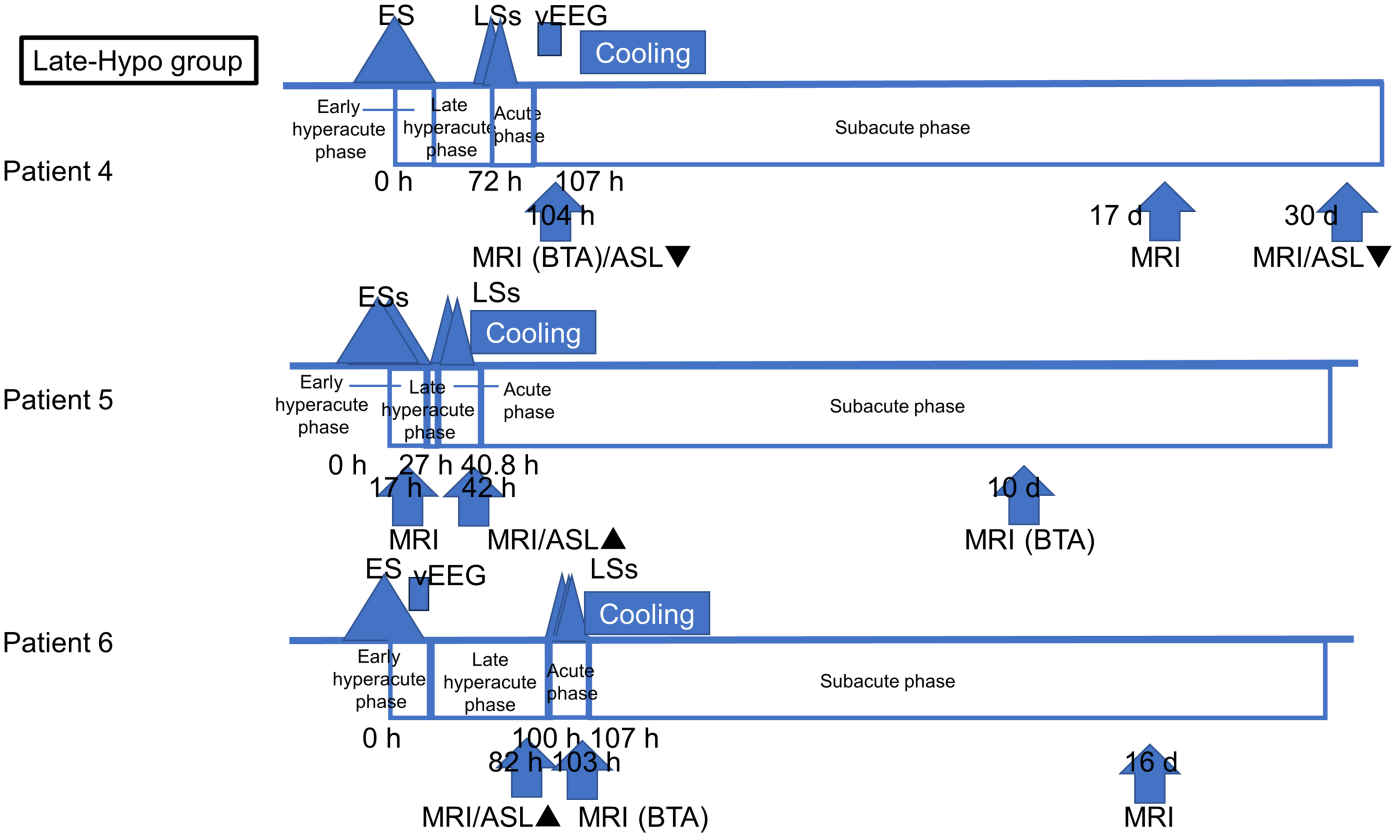
Outlines of the timing of ASL and MR imaging acquisition in each patient in the Late-Hypo group. The clinical course of AESD was divided into four phases: early hyperacute phase (within 24 h after ES/ESs), late hyperacute phase (from 24 h after ES/ESs to LSs), acute phase (within 24 h after LSs), and subacute phase (from 24 h after LSs to 30 days after ES/ESs). The timing of the initiation of LSs, cooling initiation, and image acquisition is shown in hours from the onset of ES/ESs.

All patients with AESD underwent therapeutic hypothermia during the study period. Our previous report described the methods, and all patients were cooled following a previously reported protocol (Sakata et al., 2020). Based on the timing of therapeutic hypothermia initiation, the patients were classified into two groups: patients who commenced therapeutic hypothermia, administered by an attending physician, before LS onset because of a worsening level of consciousness or continuously impaired consciousness (Early-Hypo group) and patients who started undergoing therapeutic hypothermia after LS initiation (Late-Hypo group). Following the previous report by Uetani et al. (2020), the clinical course of AESD was divided into the following phases with some modifications. Dividing the hyperacute phase into two periods in our study enabled us to compare the results of previous studies and ours. The hyperacute phase was divided into the period within 24 h after ES/ESs (early hyperacute phase) and the period from 24 h after ES/ESs to LSs in the Late-Hypo group or cooling initiation in the Early-Hypo group due to LSs masked by anaesthesia (late hyperacute phase). The acute phase is the period within 24 h after LSs in the Late-Hypo group or 3 days after cooling initiation in the Early-Hypo group. The subacute phase is the period from 24 h after LSs in the Late-Hypo group or 3 days after cooling initiation in the Early-Hypo group to 30 days after ES/ESs (Figures 1, 2).

Eight ASL images were obtained from six patients. Of these, four ASL images were obtained during the late hyperacute phase: one during the acute phase and three during the subacute phase. Figure 1 depicts the timing of ASL and MR image acquisition for each patient.

Methylprednisolone pulse therapy or immunoglobulin was not administered to any patient in the hyperacute and acute phases during this study period. This study and the prospective hypothermia plus RIPoC efficacy study on AESD were approved by the institutional review board (IRB) of St Mary’s Hospital, Fukuoka, Japan (IRB number: 23–0605 and 18–0203, respectively). All methods used were consistent with relevant guidelines and regulations. Informed consent was obtained from the patients’ legal guardians for this study and the ongoing prospective hypothermia plus RIPoC efficacy study on AESD.

### 2.2. MRI protocol

Sixteen MRI examinations (eight with 3D ASL and eight without ASL) were performed using a 3 T unit (Ingenia Elition S, Philips Healthcare. Best, Netherlands) with a 16-channel standard head coil. The MRI protocols included 3D ASL, axial T1-weighted image, T2-weighted image, fluid-attenuated inversion recovery, T2*-weighted gradient-echo, and DWI images, with or without time of flight-magnetic resonance angiography. A pseudo-continuous ASL sequence was used for evaluating brain perfusion with the following parameters: post-labeling delay, 1,500 (in patients 3–5), 1800 (in patient 1), or 2000 (in patients 2 and 6) ms; matrix, 80×96 mm; slice thickness, 8 mm; repetition time, 4,000 ms; echo time, 13 ms; flip angle, 90°; acquisition time, 120 s.

### 2.3. Image analysis

Following the analysis method previously reported by Uetani et al. (2020), two readers, a paediatrician with >5 years of experience in neuroimaging reading and a neuroradiologist, qualitatively assessed the ASL images. They compared the abnormal perfusion region with the perirolandic regions where BTA is usually spared. Scoring includes using a five-point grading system of −2, −1, 0, 1, and 2 for moderate-to-severe hypoperfusion, mild hypoperfusion, equivalent perfusion, mild hyperperfusion, and moderate-to-severe hyperperfusion, respectively. Signal change in BTA on DWI images was qualitatively assessed using a three-point grading system: 0, absent; 1, slight; and 2, apparent. The level of interobserver agreement for qualitative scored perfusion abnormality on ASL between the two readers was analysed using weighted *κ*statistics: *κ* < 0.20 = poor, 0.21–0.40 = fair, 0.41–0.60 = moderate, 0.61–0.80 = good, 0.81–0.90 = very good, and > 0.90 = excellent. The two readers reached an agreement after a discussion about the abnormal perfusion scores that differed between them.

In addition, in patients 2, 3, and 6 whose ASL images were obtained in the hyperacute phase, the two readers determined the concordance of the distribution between perfusion abnormality and BTA on the slice where BTA was most apparent in each patient using a four-pointed grading system according to a previous study (Uetani et al., 2020): grade 1, concordance rate was <25%; grade 2, concordance rate was 25–49%; grade 3, concordance rate was 50–75%; and grade 4, concordance rate was >75%.

In the previous study, the signal ratio, which is defined as the ratio of signal intensity between the cortical abnormal perfusion region and the perirolandic region, was also evaluated on each ASL image (Uetani et al., 2020). However, our study was conducted retrospectively, and we were unable to evaluate the signal ratio due to the inability to manually draw the region of interest caused by compressed stored ASL image data.

### 2.4. Data collection

The following clinical data were collected for each patient: age, sex, associated infections, first seizure duration, Glasgow Coma Scale scores 12–24 h after ES/ESs, treatment group (Early-Hypo, Late-Hypo, or Non-Hypo), timing (hours or days from ES/ESs) and findings of MR images including ASL image grading score and the concordance grade of the distribution between perfusion abnormality and BTA, electroencephalogram (EEG) or video-EEG (vEEG) findings obtained around ASL image acquisition, days when BTA was confirmed on MR images after ES/ESs, presence of diffuse lesions with injury around the perirolandic regions on MR images of at least one hemisphere, ASL image findings, serum levels of AST, ALT, lactate dehydrogenase, blood urea nitrogen, Crea, and glucose after ES/ESs, Tada scores after ES/ESs, timing of therapeutic hypothermia, and use of additional treatments, such as antiepileptic medications for seizure termination or prevention, intravenous methylprednisolone pulse therapy, or immunoglobulins (1 g/kg/dose).

## 3. Results

**Figure 3:**
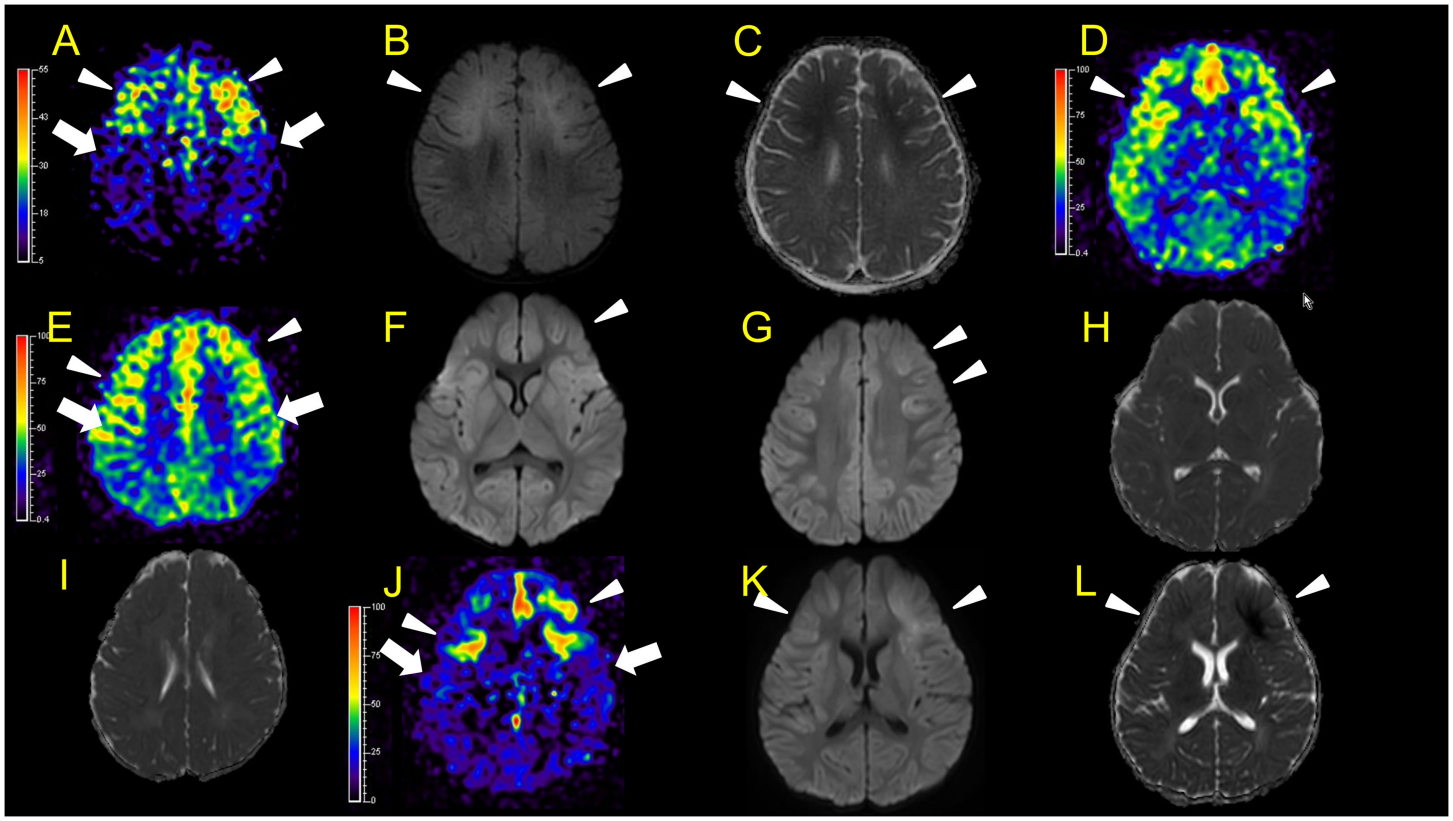
Neuroimaging findings. Color bars for ASL indicate signal intensities, not absolute perfusion values. Patient 1 **(A–C)**: ASL (post-labelling delay 1800 ms), DWI, ADC on day 7. Hyperperfusion areas (**A**: arrowheads) in bilateral frontal regions coincide with lesions in DWI and ADC (**B,C**: arrowheads). Patient 2 **(D–L)**: **D–I**, ASL (**D**,**E**, post-labelling delay 2000 ms), DWI **(F,G)**, ADC **(H,I)** at 31 h after ESs (**E,G,I** are higher slices of **D,F,H**). There are hyperperfusion areas in the bilateral prefrontal regions (bilateral middle frontal lobes and left medial prefrontal cortex; **D**: arrowheads) compared to perirolandic regions (arrows). There are slight abnormal changes in DWI (**F, G**: arrowheads) in the left frontal region, which were not accompanied by apparent diffusion coefficient abnormalities **(H,I)**. **(J–L)** ASL (post-labelling delay 2000 ms), DWI, and ADC at 82 h after ESs. ASL (**J**: arrowheads) exhibited more prominent hyperperfusion areas than the first ASL image compared to perirolandic regions (**J**: arrows). On DWI and ADC, BTA appeared on bilateral frontal regions (**K,L**, arrowheads).

**Figure 4:**
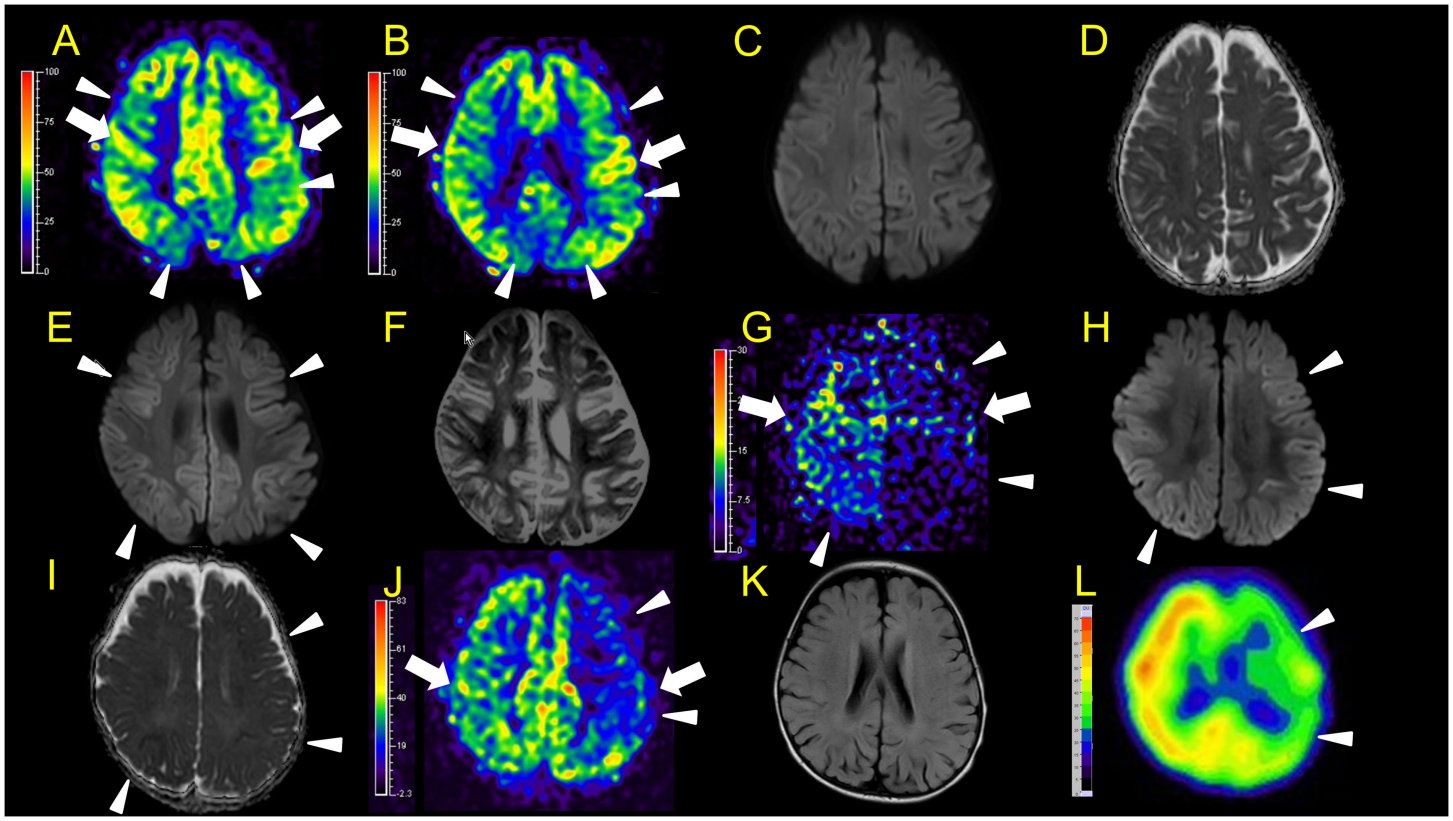
Neuroimaging findings. Color bars for ASL and SPECT indicate signal intensities, not absolute perfusion values. Patient 3 **(A–F)**: **A, B**, ASL (**A and B**, post-labelling delay 1,500 ms, **B** is a lower slice of **A**), DWI **(C)**, ADC **(D)** at 46 h after ESs. ASL (**A**: arrowheads) revealed hypoperfusion areas compared to perirolandic regions (arrows) in the bilateral frontal, left parietal, and bilateral occipital regions without DWI or ADC abnormalities **(C,D)**. **E,F**, DWI, and ADC on day 12. Hypoperfusion areas in bilateral frontal and parietal lobes in ASL at 46 h after ESs (**A**: arrowheads) coincide with BTA (**E**: arrowheads) on day 12. Patient 4 **(G–L)**: **G–I**, ASL (post-labelling delay 1,500 ms), DWI, ADC at 104 h after ESs. ASL image obtained in the subacute phase revealed hypoperfusion in the right frontal and parietal regions, and the entire left hemisphere (**G**: arrowheads) compared to the right perirolandic region (arrows). Broad hypoperfusion areas in the left hemisphere were seen. DWI and ADC revealed slight BTA in the left frontal and bilateral parietal regions (**H,I**: arrowheads). **(J,K)** ASL (post-labelling delay 1,500 ms) and FLAIR on day 30. **L**, SPECT (with 99mTc-ECD) on day 43. ASL images and SPECT acquired in the subacute phase persistently showed hypoperfusion areas in the left hemisphere (**J,L**: arrowheads) compared to the right perirolandic region (**J**: arrow) without abnormal FLAIR findings.

**Figure 5:**
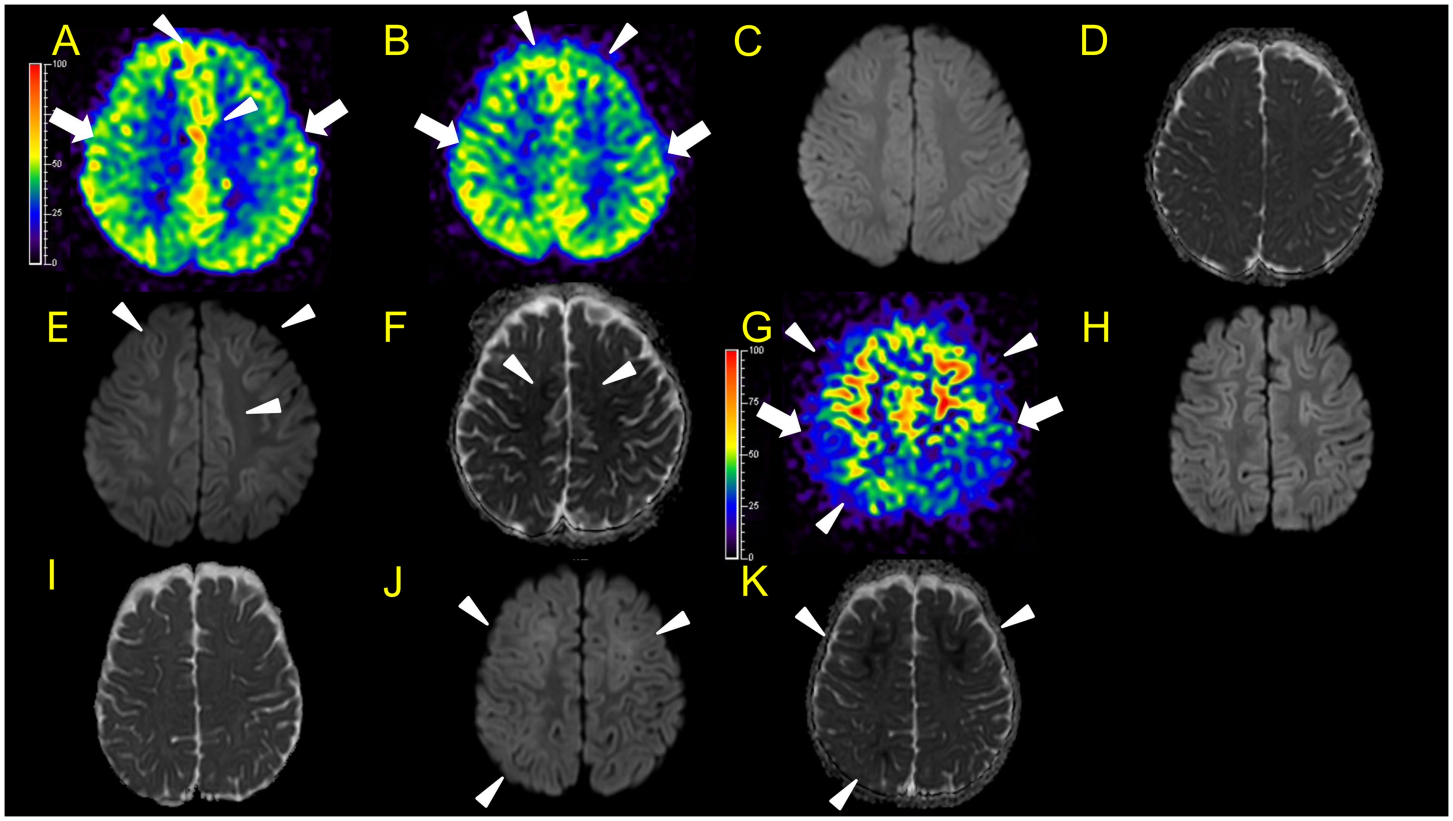
Neuroimaging findings. Colour bars for ASL indicate signal intensities, not absolute perfusion values. Patient 5 **(A–F)**: **A–D**, ASL (**A,B**, post-labelling delay 1,500 ms), DWI, ADC at 42 h after ESs. ASL revealed hyperperfusion in the bilateral frontal regions (**A,B**: arrowheads) compared to perirolandic regions (arrows), while DWI or ADC showed no abnormalities **(C,D)**. Although there is the possibility that hypoperfusion areas exist in the left lateral frontal lobe, we were unable to evaluate the signal ratio between the cortical abnormal perfusion region and the perirolandic region due to the technical reason mentioned in the materials and methods section. **(E,F)** DWI and ADC on day 10. Hyperperfusion areas in bilateral frontal lobes in ASL at 42 h from ESs coincide with BTA on day 10 (**E,F**: arrowheads). Patient 6 **(G–K)**, **G–I**, ASL (post-labelling delay 2000 ms), DWI, ADC at 82 h after ESs. **(J,K)** DWI and ADC at 103 h. Hyperperfusion areas in bilateral frontal lobes and right parietal lobe in ASL at 82 h after ESs (**G**: arrowheads) compared to the perirolandic region (arrows), while DWI or ADC showed no abnormalities, which coincide with BTA at 103 h (**J,K**: arrowheads).

Eight ASL images were obtained during the hospital stay of six out of the 12 patients with AESD admitted to our hospital during the study period. Figures 3–5 display all eight ASL images, relevant MR images, and other neuroimages. Table 1 and the supplementary table present the background information. Table 2 presents a summary of MRI findings, including the eight ASL images in six patients. The level of interobserver agreement for qualitative scored perfusion abnormality on ASL was very good (κ = 0.82).

**Table 1:** Clinical characteristics of six patients with acute encephalopathy with biphasic seizures and late reduced diffusion (AESD).

Patient number	Treatment option (Early-Hypo, Late-Hypo group)	Age (months)	Sex (female or male)	First seizure duration (minutes)	GCS between 12 and 24 h after ES/ESs	The duration between ES/ESs and the initiation of LSs (h)	BTA (on MRI) timing (days from ES/ESs day)	Distribution of the brain lesion on MRI (1 unilateral, 2 bilateral frontal, or 3 others)	Presence of diffuse lesions with injury around the perirolandic regions on MR images 1 Yes, 2 No	Tada score	Yocochi score
1	Early hypo	10	Female	54	6		7	2	1	7	7
2	Early hypo	15	Female	97	9		3	2	1	5	7
3	Early hypo	21	Male	5	10		12	2 + 3	1	3	3
4	Late hypo	11	Male	2	15	72	3	3	1	?*	?*
5	Late hypo	16	Male	33	11	27	10	2	1	4	1
6	Late hypo	20	Male	145	12	100	4	2 + 3	1	7	6

*Blood test was not performed after ES/ESs.

**Table 2:** Summary of MRI findings in six patients with acute encephalopathy with biphasic seizures and late reduced diffusion (AESD).

Patient number	Hyperacute phase						Acute phase			Subacute phase		
	Early hyperacute phase			Late hyperacute phase								
	Timi ng	ASL grade	BTA grade	Timing	ASL grade	BTA grade	Timing	ASL grade	BTA grade	Timing	ASL grade	BTA grade
Early-hypo												
1	N/A	N/A	N/A	N/A	N/A	N/A	N/A	N/A	N/A	7 d	2*	2
2	N/A	N/A	N/A	31 h/82 h	1/2	0/2	N/A	N/A	N/A	20 d	N/A	0
3	N/A	N/A	N/A	46 h/68 h	-1/N/A	0/0	N/A	N/A	N/A	12 d	N/A	2
Late-hypo												
4	N/A	N/A	N/A	N/A	N/A	N/A	N/A	N/A	N/A	104 h/17 d/30 d	−2/N/A/−1*	2/0/0
5	17 h	N/A	0	N/A	N/A	N/A	42 h	1*	0	10 d	N/A	2
6	N/A	N/A	N/A	82 h	+2	0	103 h	N/A	2	16 d	N/A	0

The MRI findings were presented based on the timing of the acquisition.

The clinical course of AESD was divided into four phases based on the imaging timing: The hyperacute phase was divided into the period within 24 h after ES/ESs (early hyperacute phase) and the period from 24 h after ES/ESs to LSs in the Late-Hypo group or cooling initiation in the Early-Hypo group due to LSs masked by anaesthesia (late hyperacute phase). The acute phase is the period within 24 h after LSs in the Late-Hypo group or 3 days after cooling initiation in the Early-Hypo group. The subacute phase is the period from 24 h after LSs in the Late-Hypo group or 3 days after cooling initiation in the Early-Hypo group to 30 days after ES/ESs.

*These images were obtained after the remote ischaemic post-conditioning procedure.

The timing of the MRI is presented in hours (h) or days (d) for ES/ESs.

The scores were obtained utilising a five-point grading system, including − 2, −1, 0, 1, and 2 for moderate-to-severe hypoperfusion, mild hypoperfusion, equivalent perfusion, mild hyperperfusion, and moderate-to-severe hyperperfusion, respectively.

The assessment of signal change in BTA on diffusion-weighted imaging (DWI) was qualitatively assessed using a three-point grading system: 0, absent; 1, slight; and 2, apparent.

N/A, not applicable; BTA, bright-tree appearance.

In patient 1, ASL in the subacute phase (on day 7) revealed that hyperperfusion areas (Figure 3A) in the bilateral frontal regions coincided with lesions on DWI and ADC (Figures 3B,C) obtained on the same day.

**Figure 6:**
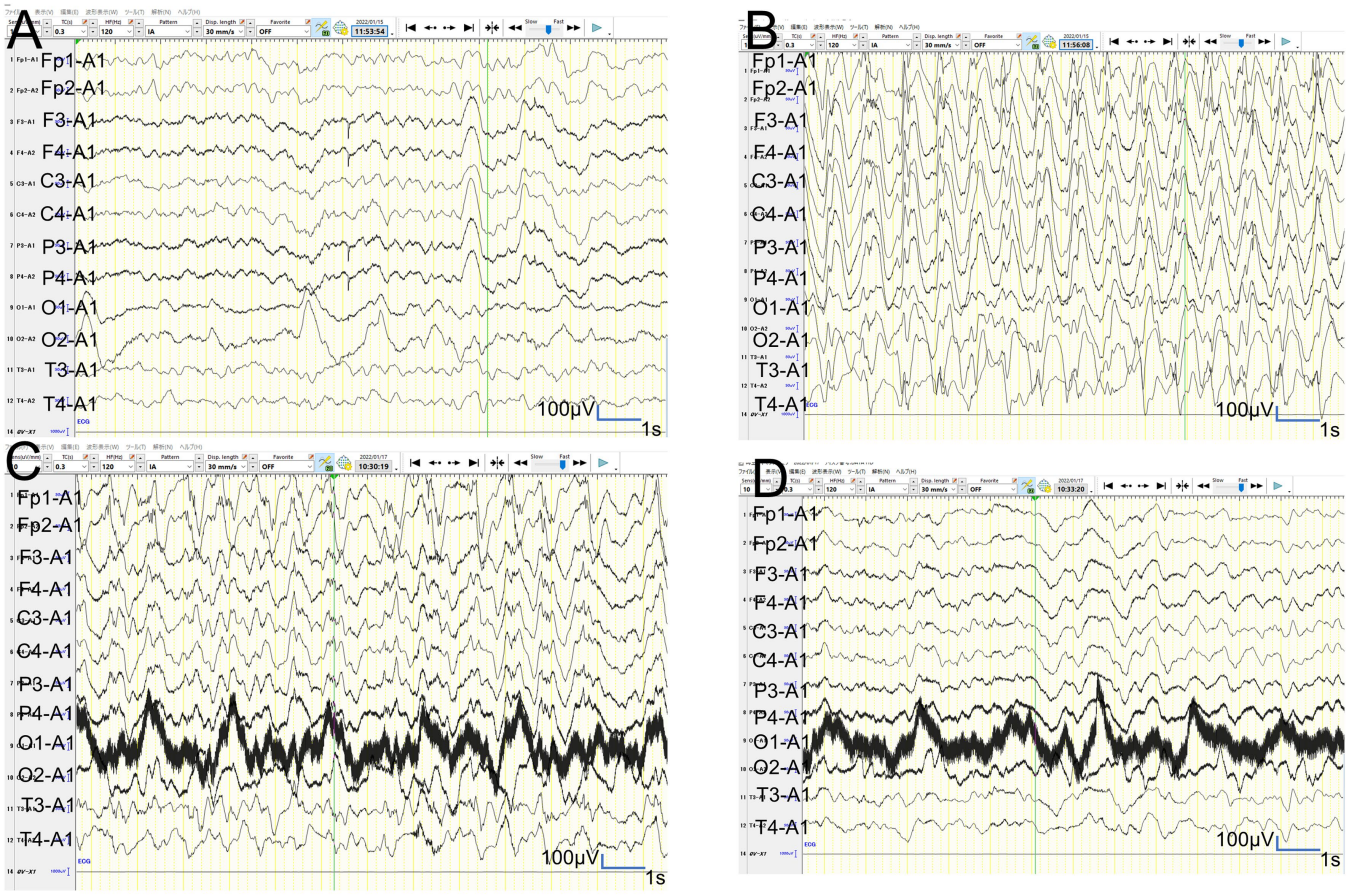
Video-EEG findings. **(A,B)**: In patient 2, the vEEG recorded for 2 h just before MRI revealed generalized slowing with one electrographic seizure event lasting for 90 s 1 h before MRI. **(C,D)**: In patient 2, the vEEG recorded for 2 h until 2 h before the second ASL image revealed one electrographic seizure in bilateral frontal regions lasting 14 min (Fp1 and Fp2).

In patient 2, the first ASL image was obtained in the late hyperacute phase (31 h from ES). It revealed hyperperfusion areas in the bilateral prefrontal regions (bilateral middle frontal lobes and left medial prefrontal cortex; Figures 3D,E) with slightly abnormal changes on DWI in the left frontal region, which were not accompanied by ADC abnormalities (Figures 3F–I). The vEEG recording, conducted for 2 h before MRI, revealed generalized slowing with one electrographic seizure event lasting for 90 s 1 h before MRI (Figures 6A,B). Thiopental was administered for sedation during the MR image acquisition, as described above. The second ASL image was also obtained in the late hyperacute phase (82 h from ES, Figure 3J) because drowsiness persisted even on the fourth day from ES, although the patient could sit alone with truncal instability. This image exhibited more prominent hyperfusion areas than the first ASL image. On DWI and ADC, BTA appeared on the bilateral frontal regions (Figures 3K,L). The vEEG recording, conducted for 2 h until 2 h before the second ASL image, revealed one electrographic seizure in the bilateral frontal regions lasting 14 min (Fp1 and Fp2) (Figures 6C,D). Thiopental was administered again for sedation during the MR image acquisition.

**Figure 7:**
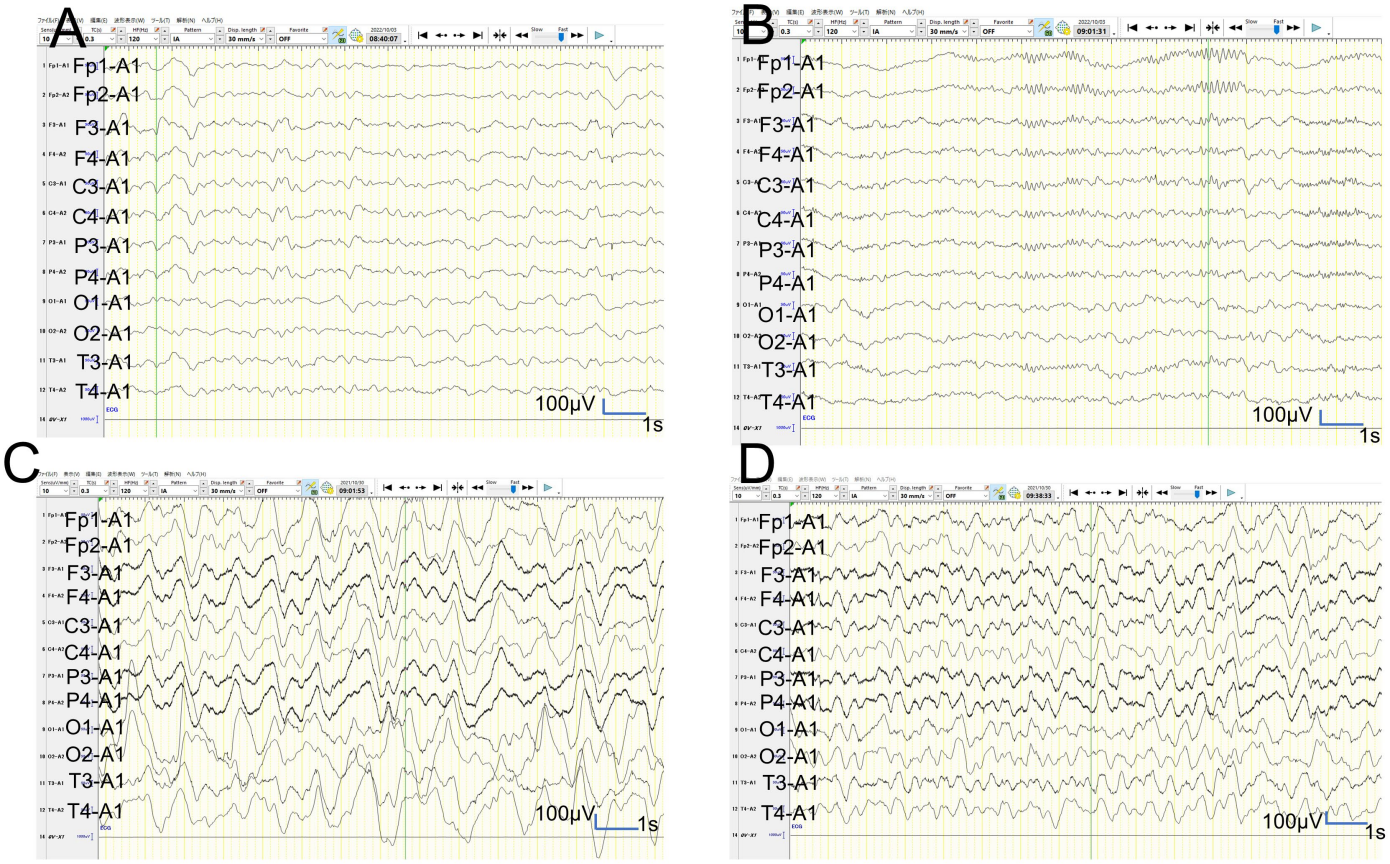
Video-EEG findings. **(A,B)**: In patient 3, the 6-h vEEG recording started at 15 h after ASL acquisition revealed no electrographic seizure. **(C,D)**: In patient 6, the vEEG obtained for 6 h from 2.5 h after ES revealed generalized high voltage slow waves without electrical seizures.

In patient 3, the ASL image was obtained in the late hyperacute phase (at 46 h from ES). It revealed hypoperfusion areas in the bilateral frontal, left parietal, and bilateral occipital regions (Figures 4A,B) without DWI or ADC abnormalities (Figures 4C,D). The 6-h vEEG recording, started 15 h after ASL image acquisition, revealed no electrographic seizure (Figures 7A,B).

In patient 4, the first ASL image obtained in the subacute phase revealed hypoperfusion in the right frontal and parietal regions, and the entire left hemisphere (Figure 4G). DWI and ADC revealed slight BTA in the left frontal and bilateral parietal regions (Figures 4H,I). Hypoperfusion in the entire left hemisphere was initially considered an artefact. However, we concluded that it was not all caused by an artefact because subsequent ASL images and regional CBF measurements, using the Patlak plot method and SPECT with technetium 99 m-ethyl cysteinate dimer, acquired in the subacute phase consistently showed the same results (Figures 4J,L) without FLAIR showing abnormal findings (Figure 4K).

In patient 5, ASL obtained in the acute phase revealed hyperperfusion in the bilateral frontal and parietal regions without DWI or ADC abnormalities (Figures 5A–D). Hyperperfusion areas in bilateral frontal lobes in ASL at 42 h from ESs coincide with BTA on Day 10 (Figures 5E,F). Although two readers evaluated this patient’s ASL images as hyperperperfusion, there is a possibility that hypoperfusion areas exist in the left lateral frontal lobe. Regarding this matter, we were unable to evaluate the signal ratio between the cortical abnormal perfusion region and the perirolandic region due to the inability to manually draw the region of interest caused by compressed stored ASL image data as mentioned in the Materials and Methods section.

In patient 6, the patient was transiently intubated owing to respiratory distress following a 2-h status febrile seizure, which was terminated with the administration of diazepam, midazolam, and fosphenytoin. vEEG recording, conducted for 6 h starting from 2.5 h after ES, revealed generalized high voltage slow waves without electrical seizures (Figures 7C,D). The patient was extubated 6 h later, and drowsiness gradually improved. On the third day, the patient started sitting alone with truncal instability and could play with toys using both hands on the fourth day. The ASL image acquisition at 82 h after ES revealed hyperperfusion areas in bilateral frontal lobes without DWI or ADC abnormalities (Figures 5G–I). No vEEG was performed around the ASL image acquisition. Hyperperfusion areas in the bilateral frontal lobes and right parietal lobe in ASL at 82 h after ESs coincide with BTA at 103 h (Figures 5J,K).

No ASL images were obtained in the early hyperacute phase. Four ASL images were obtained from three patients (patients 2, 3, and 6) in the late hyperacute phase. Among these, three ASL images from two patients (patients 2 and 6) exhibited hyperperfusion (Figures 3D,E,J, 5G). One ASL image was obtained in the acute phase, showing hyperperfusion in patient 5 (Figures 5A,B). Three ASL images were obtained in the subacute phase, one showing hyperperfusion (patient 1, Figure 3A) and the other two showing hypoperfusion (patient 4, Figures 4G,J).

In two patients with AESD (patients 2 and 6), the hyperperfusion regions observed on ASL in the late hyperacute phase (Figsures 3D,E,J, 5G) corresponded to the “BTA” in the subsequent MR images (Figures 3K,L, 5J,K). In patient 2, the concordance of the distribution between perfusion abnormality and BTA was evaluated as grade 3 and 4 by two readers, respectively. In patient 6, the concordance was evaluated as grade 4 by both readers. In one patient with AESD (patient 3), the hypoperfusion regions observed on ASL in the late hyperacute phase (Figures 4A,B) slightly corresponded to the “BTA” in the subsequent MR images (Figures 4E,F). The concordance of the distribution between perfusion abnormality and BTA was evaluated as grade 2 and 1 by the two readers, respectively. Neither of these patients exhibited any episodes suggestive of clinical seizures between ES and the image acquisition.

## 4. Discussion

This study aimed to investigate the ASL findings in patients with AESD on the timing of ASL image acquisition and discovered that two exhibited hyperperfusion regions and another patient exhibited hypoperfusion regions among three patients who underwent ASL imaging during the period from 24 h after ES/ESs to LSs in the Late-Hypo group or cooling initiation in the Early-Hypo group (late hyperacute phase).

Previous studies have reported decreased CBF within 24 h after ES/ESs in patients with AESD (Kuya et al., 2017; Yokoyama et al., 2017; Uetani et al., 2020). Transient hypoperfusion for a couple of hours after a seizure on ASL or SPECT has been observed in patients with epilepsy, and hypoperfusion lasting more than that period on ASL in AESD might indicate irreversible neuronal damage (Nelissen et al., 2006; Telischak et al., 2015; Gaxiola-Valdez et al., 2017; Yokoyama et al., 2017). This finding corresponded to the findings of patient 3 in our study. Moreover, hyperperfusion after LSs in patients with AESD has been previously reported (Kuya et al., 2017; Uetani et al., 2020). In a case report of a patient with AESD, the MR image obtained 1 day before LS onset showed hyperperfusion in the bilateral posterior frontal areas on ASL, high signal intensity on DWI images, and no ADC abnormalities. The authors speculated that the affected area requires more glucose and oxygen, causing compensatory regional hyperperfusion. Moreover, insufficient supply to the hyperactive cortical area resulted in pathophysiological changes that cause cytotoxic oedema (Morita et al., 2021).

In our study, two patients (patients 2 and 6) showed hyperperfusion in the late hyperacute phase. Patient 2 showed abnormal changes on DWI in the right frontal region at the same time, which were not accompanied by ADC abnormalities (Figures 3F–I). This corresponded to the findings of the patient with AESD who exhibited hyperperfusion on ASL, high signal intensity on DWI images, and no ADC abnormalities before LS onset, as reported by Morita et al., which is described above. Meanwhile, patient 6 showed hyperperfusion areas without abnormal change on DWI or ADC on the ASL image obtained 82 h after ES, which was 18 h before LS onset. This finding has not been previously reported, possibly because the timing of ASL acquisition between ES/ESs and LSs in this patient (82 h after ESs) was later than that previously reported by Uetani et al. (8.5–22 h after ESs), Kyuya et al. (21 h after ESs), and Yokoyama et al. (18 h after ESs). However, our timing was earlier than that reported by Morita et al. (5 days after ESs) (Kuya et al., 2017; Yokoyama et al., 2017; Uetani et al., 2020; Morita et al., 2021).

The mechanisms underlying AESD have not been clarified; however, evidence suggests that it may be attributed to late neuronal death triggered by extracellular glutamate stimulation during ES/ESs (Takanashi et al., 2006). Cerebral hyperperfusion on ASL, performed on an average of 4.6 days after the anoxic episode, is also observed in patients with anoxic injury. The mechanism for this hyperperfusion might stem from a loss of autoregulation secondary to the injury (Pollock et al., 2008). Similarly, the findings in our cases may support the idea that the abnormal perfusion around LSs in patients with AESD is caused by the loss of autoregulation in CBF following dysfunction of astrocytes or microglia after the first status epilepticus (Kuya et al., 2017).

Transient hypoperfusion on SPECT after simple seizures has been reported (Leonhardt et al., 2005). Thus, previous studies have recommended obtaining ASL images 4 h after seizures when AESD is suspected (Yokoyama et al., 2017). In addition to the ASL image acquisition at approximately 4 h after ES/ESs, repeated ASL image acquisition 3–4 days after ES/ESs might be useful for early diagnosis of AESD before LS onset because abnormal findings are not typically observed on DWI in patients with AESD before LS onset, making early diagnosis challenging and delaying intervention in these populations. Moreover, the proposed AESD predictive score showed a low positive predictive value of 47% (Yokochi et al., 2016). However, this is the first report to show hyperperfusion before the appearance of DWI or ADC abnormalities, and LSs. We were unable to ascertain whether CBF increased in patients with AESD after transiently decreased CBF after ES/ESs, and if so, the exact time points when CBF increased in patients with AESD after transiently decreased CBF after ES/ESs. In addition, the transition from hypoperfusion to hyperperfusion before LS onset in patients with AESD might depend on disease severity because a previous study reported a moderate negative association between worse outcomes and the duration between ES/ESs and LSs (Sakata et al., 2020). This might explain why patient 2 in our study showed high signal intensity on DWI images accompanied by hyperperfusion 31 h after ES/ESs, which was earlier than the ASL image timing in patient 6. Therefore, future prospective studies should continuously analyse CBF using a device, such as near-infrared spectroscopy in patients with AESD.

Regarding the concordance of the distribution between perfusion abnormality and BTA in patients 2, 3, and 6, whose ASL images were obtained in the hyperacute phase, the concordance rate of patient 2 was evaluated as grade 3 and 4 by two different readers, and that of patient 3 was evaluated as grade 2 and 1, although that of patient 6 was evaluated as grade 4 by both readers. The fact that the concordance rates of all these three cases were not grade 4 may indicate the presence of both hypoperfusion and hyperperfusion areas within the same images and also in the same patients. These findings might also be explained by two hypotheses. The first one is that only the regions related to subclinical seizures exhibited hyperperfusion, and other regions exhibited hypoperfusion. The second one is that the initial hypoperfusion areas may have changed to hyperperfusion, and the timing may vary in regions in each patient. Clustered subclinical seizures before LS onset have been previously reported in a patient with AESD (Komatsu et al., 2010). Our patient 2 had electrographic seizures when the first and second ASL images, obtained at 31 h and 82 h from ES, respectively, showed hyperperfusion. In contrast, patient 3 had no electrographic seizures during the 6-h vEEG recording that started at 15 h after ASL acquisition, suggesting the absence of electrographic seizures when the first ASL image showed hypoperfusion at 46 h from ES. Based on our findings and previously reported findings, lesions with hypoperfusion shortly after ES/ESs may transition to hyperperfusion 3–5 days after ES/ESs (1–2 days before LS onset), accompanied by electrographic seizures. Eventually, these patients may develop “BTA” at LS onset in AESD. Hyperperfusion on ASL before showing DWI or ADC abnormalities or LSs may be associated with electrographic seizures. These events might be attributed to the dysfunction of astrocytes or microglia after the first status epilepticus leading to compensatory regional hyperperfusion to meet glucose and oxygen demands in the lesions because the perfusion-weighted imaging by dynamic susceptibility contrast-enhanced MRI in patients with temporal or parietal lobe epilepsy revealed that postictal relative hypoperfusion in the hippocampus appears to be associated with the cessation of neuronal ictal discharge. In contrast, postictal hyperperfusion in the parahippocampal gyrus lags and may reflect increased metabolism to restore the interictal state of neuronal excitability (Leonhardt et al., 2005). The relationship between ASL image findings and electrographic seizures in patients with AESD must be clarified in larger prospective studies in the future.

This study has some limitations. There are only four ASL images from three cases obtained during the hyperacute phase because the timing of ASL image acquisition varied among patients owing to technical reasons or patient conditions, such as the initiation of therapeutic hypothermia. Second, although two readers compared the abnormal perfusion region with the perirolandic regions where BTA is usually spared following the previous report by Uetani et al. and reported each image either with hyperperfusion or hypoperfusion abnormalities, we might have missed detailed perfusion changes, such as a mixture of hyperperfusion and hypoperfusion in some cases as described above. In some cases whose unilateral or bilateral perirolandic regions may be involved, like in patient 4 in this study, the comparison would be difficult and may be misinterpreted. This study was also conducted using the data from the ongoing hypothermia plus RIPoC efficacy study in AESD. Therefore, vEEG timing was not precisely determined based on a protocol and varied among patients, and the vEEG recording time in each patient was not long enough to make any conclusion about the effect of electrographic seizures on ASL images. In addition, electrographic seizures during the MR image acquisition may not be completely ruled out as the cause of the increased blood flow although thiamylal was administered as the sedative for all patients during the MR image acquisition.

In conclusion, despite the limitations of extrapolating findings from a few cases, our results suggest that blood flow increases in some areas in some patients with AESD before showing DWI or ADC abnormalities and LSs, while blood flow decreases in some patients, such as previously reported or in patient 3 in this study. In addition, there may exist areas of mixing of hypoperfusion and hyperperfusion areas at the same time in the same patient. This increase in blood flow may be associated with electrographic seizures and the loss of autoregulation in CBF owing to hypoxia caused by status epilepticus, which may play a crucial role in the underlying mechanisms of AESD. Future prospective studies should investigate the relationship among sequential CBF changes, ASL and MRI findings, and EEG findings in patients with AESD.

## Data availability statement

The original contributions presented in the study are included in the article/Supplementary material, further inquiries can be directed to the corresponding author.

## Ethics statement

The studies involving humans were approved by the institutional review board of St Mary’s Hospital, Fukuoka, Japan. The studies were conducted in accordance with the local legislation and institutional requirements. Written informed consent for participation in this study was provided by the participants’ legal guardians/next of kin. Written informed consent was not obtained from the individual(s) for the publication of any potentially identifiable images or data included in this article because There is no data that can identify individuals.

## Author contributions

GK, KT, and TM contributed to the study design, data curation, interpretation of the results, and manuscript writing. GK, KT, YK, KS, HS, TY, and TM contributed to the research. All authors contributed to manuscript revision, read, and approved the submitted version.

## Funding

This study was supported by a grant from the MHLW Research programme on rare and intractable diseases (grant number JPMH23FC1013).

## Conflict of interest

The authors declare that the research was conducted in the absence of any commercial or financial relationships that could be construed as a potential conflict of interest.

## Publisher’s note

All claims expressed in this article are solely those of the authors and do not necessarily represent those of their affiliated organizations, or those of the publisher, the editors and the reviewers. Any product that may be evaluated in this article, or claim that may be made by its manufacturer, is not guaranteed or endorsed by the publisher.

## Figures and Tables

**Table:** 

ASL	Arterial spin labelling
AESD	Acute encephalopathy with biphasic seizures and late reduced diffusion
ES	Early seizure
LSs	Late seizures
ADC	Apparent diffusion coefficient
DWI	Diffusion-weighted imaging
MRI	Magnetic resonance imaging
BTA	Bright-tree appearance
CBF	Cerebral blood flow
SPECT	Single photon emission computed tomography
RIPoC	Remote ischaemic post conditioning
AST	Aspartate transaminase
ALT	Alanine transaminase
Crea	Creatinine
vEEG	Video electroencephalogram
