# Supplementary material for: Arterial spin labeling image findings in the acute phase in paediatric patients with acute encephalopathy with biphasic seizures and late reduced diffusion
Source: Front Neurosci. 2023 Sep 19;17:1252410. doi: 10.3389/fnins.2023.1252410 (PMC10545960; doi:10.3389/fnins.2023.1252410)
Supplement: Supplementary file 1 [file Table_1.DOCX]

**Supplementary Table** Additional clinical characteristics of six patients with AESD.

|  | AST | ALT | LDH | BUN | Crea | Glu | Associated infection | The duration between the first seizure and the initiation of therapeutic hypothermia (hrs) | The duration between the second phase and the initiation of the therapeutic hypothermia (hrs) |
| --- | --- | --- | --- | --- | --- | --- | --- | --- | --- |
| 1 | 107 | 42 | 323 | 16.3 | 0.33 | 410 | Unidentified | 14.3 |  |
| 2 | 87 | 39 | 483 | 36.8 | 0.34 | 4 | Unidentified | 84 |  |
| 3 | 36 | 11 | 344 | 8 | 0.22 | 99 | Unidentified | 69 |  |
| 4 | * | * | * | * | * | * | Adenovirus14.3 | 107 | 35 |
| 5 | 52 | 21 | 363 | 10.8 | 0.3 | 126 | Unidentified | 42 | 19 |
| 6 | 208 | 68 | 589 | 1 | 0.38 | 313 | Echovirus 22 | 107 | 6.5 |

*Blood test was not done after the first seizure
